# Bacterial Colonization in Double J Stent and Bacteriuria in Post-Renal Transplant Patients

**DOI:** 10.7759/cureus.27508

**Published:** 2022-07-31

**Authors:** Sadiq Abu, Stephen O Asaolu, Martin C Igbokwe, Olalekan O Olatise, Kenenna Obiatuegwu, Uzodimma E Onwuasoanya, Adefola R Adetunbi

**Affiliations:** 1 Department of Surgery/Urology Unit, Zenith Medical and Kidney Centre, Abuja, NGA; 2 Department of Urology, Milton Keynes University Hospital, Milton Keynes, GBR; 3 Department of Clinical Research, Zenith Medical and Kidney Centre, Abuja, NGA; 4 Department of Medicine/Nephrology Unit, Zenith Medical and Kidney Centre, Abuja., NGA; 5 Department of Surgery/Urology Division, Federal Medical Centre, Jabi, Abuja, NGA; 6 Department of Surgery, Zenith Medical and Kidney Centre, Abuja, NGA

**Keywords:** antibiotic resist, antibiotic sensitivity pattern, renal transplant, bacteriuria, double j stents, bacterial colonization

## Abstract

Background and objective

Urinary tract infections (UTI) in kidney transplant recipients can cause significant morbidity and negatively impact both, graft and patient survival. Ureteric stenting in renal transplantation is aimed at reducing the risks of complications like ureteric leak and stenosis. Ureteric stents are not without their potential complications which may include UTI. We aimed to compare urine bacteriology and bacterial colonization of DJ stent following kidney transplantation, and to establish antimicrobial susceptibility to guide the choice of empirical antibiotics in the event of UTI in post-transplant patients with DJ stent.

Materials and methods

This was a prospective study carried out over a year period (February 2020 to January 2021). Eighty post-renal transplant patients with indwelling ureteral stents were recruited for the study. An early morning midstream urine sample was taken for analysis from consenting patients that met the inclusion criteria. All stents were removed via rigid cystoscopy and the distal end of the stent (4cm) was cut off and put in a sterile bottle for microbiological analysis. Sensitivity and resistance were tested against a panel of 19 antibiotics on all microbial isolates. Results were considered statistically significant when p < 0.05.

Results

The mean age of the patients was 47.9+ 12.1 years. Male patients were 60 (75%) while 20 (25%) were females. Fifty-one (52%) patients had hypertension while 25 (26%) had diabetes mellitus. Hypertension and diabetes were noted in 20 (21%) patients while only one patient (1%) had HCV. Prior to renal transplantation, patients had negative urine cultures. The majority of the patients (76, 95%) had their stent retrieved after 4 weeks, 2 (2.5%) of them had stents retrieved after 2 weeks, and 2 (2.5%) had stents retrieved after 8 weeks. There was a significant association between the duration of stent and stent colonization (p=0.031).

No organism was cultured in both the urine and stent in 13 (14.4%) patients. Nine (10%) had positive stent culture with a negative urine culture while 5 (5.6%) had positive urine culture with a negative stent culture. The same organism was noted in both urine and stent in 58 (64.4%) of patients while different organisms were cultured in 5 (5.6%) of the patients. Escherichia coli was the most common organism cultured in the urine of 38 (65.5%) patients and 36 (58.1%) stents, respectively.

The sensitivity pattern shows that the organisms were more susceptible to nitrofurantoin and gentamicin, and resistant to tetracycline and ceftriaxone. Tigecycline showed good susceptibility and poor resistance.

Conclusion

This study shows that stent colonization was slightly higher than urine bacteriology, with both demonstrating similar microbiological patterns. Selection of the initial empiric treatment should be based on local epidemiological data. Initial therapy should be de-escalated to the most narrow-spectrum antibiotics to complete the course of therapy once culture and sensitivity data is available. Antibiotics stewardship will help in reducing the trend of MDR pathogens.

## Introduction

Many people all over the world are affected by end-stage renal disease (ESRD) and its prevalence in Nigeria is increasing [1,2]. Kidney transplantation (KT) is recommended as the gold standard out of all available renal replacement therapies for patients living with ESRD [3]. In addition to being cost-effective, KT is superior to hemodialysis because the quality of life and life expectancy of ESRD patients increases significantly after the procedure [3,4].

A lot of advances have been made in the field of post-transplant immunosuppressive therapy leading to improved graft outcomes. Despite these advances, complications due to infections are yet to improve, with the most common infection site being the urinary tract. Urinary tract infection (UTI) has been reported to occur in up to 60% of post-renal transplant patients in the first year following transplant [5,6]. Apart from immunosuppressives, other risk factors attributed to this are urological abnormalities, vesicoureteral reflux (VUR), invasive, diagnostic, and therapeutic procedures involving the urinary tract, and the presence of ureteric stents [5]. For post-transplant patients, UTI can be a source of concern; it can negatively impact graft survival and overall health outcomes. Ureteric stenting like Double J stent (DJ stent) is a procedure commonly performed during renal transplantation in order to bring down the likelihood of urologic complications such as ureteric leak and stenosis. However, ureteric stents are not without their potential complications which include bacteria colonization and urinary tract infections [7,8]. This is why most kidney transplant surgeons advise against using ureteric stents routinely for all patients and prefer the use of stents on a case-by-case basis [7,8].

DJ stents are used for all kidney transplant recipients in our center and it is removed at 4-weeks post-transplant. There is a paucity of data on DJ stent colonization in post-transplant patients in sub-Saharan Africa despite its routine use in renal transplant patients.

In this study, we aimed to compare urine bacteriology and bacteria DJ stents colonization following kidney transplantation and to establish antimicrobial susceptibility to guide the choice of empirical antibiotics in the event of UTI in post-transplant patients with DJ stents.

## Materials and methods

This was a prospective study of post-renal transplant patients that had ureteric stents, and who came for its removal 4 weeks after the transplant. The research participants were consecutively recruited into the study. Convenience sampling method was used to recruit patients for the study. A total of 80 patients from our institution were enrolled in this study over a year period between February 2020 and January 2021 after the approval from the institutional ethics review board. The aim of the study was explained to all the patients and consent was obtained.

Exclusion criteria included patients with urine leaks, bladder outlet obstruction and other vascular complications, and patients with allograft dysfunction. All the patients were placed on the same immunosuppressant regime that consisted of different combinations of mycophenolate mofetil, tacrolimus, sirolimus, cyclosporin, and steroids. All patients received co-trimoxazole (80 mg trimethoprim/400 mg sulfamethoxazole) and nystatin 500,000 units once daily for pneumocystis pneumonia and fungal prophylaxis respectively for six months. However, no specific drug was given as prophylaxis against the UTI.

All stents were removed via rigid cystoscopy and the distal end of the stent (4 cm) was cut off and put in a sterile bottle for microbiological analysis. Participants were adequately counseled on the urine collection technique and taken to the hospital laboratory restroom early in the morning where midstream urine was obtained into a sterile bottle. Collected urine was centrifuged and prepared on a slide and microscopy was done. 

Blood agar and eosin methylene blue agar was used to inoculate the urine collected. The agar plates were incubated for 48 hours at 37^0^C. Quantitative evaluation was carried out on the microorganisms that the agar produced. The bacteria were then cultured and identified using the conventional method in the microbiology department of the hospital. Sensitivity and resistance was tested against a panel of 19 antibiotics on all microbial isolates The sensitivity of the antibiotics was quantified by the measurement of their individual zones of inhibition. The handling of all the specimens was according to the Standard Operating Procedures of the Clinical Microbiology Laboratory of the hospital.

All analyses were performed using IBM SPSS Statistics for Windows, Version 21 (Armonk, NY: IBM Corp). Simple frequency and percentages were used to present relevant data, while chi-square was used for testing the relationship between categorical variables. A *p*-value of < 0.05 was considered statistically significant.

## Results

**Figure 1:**
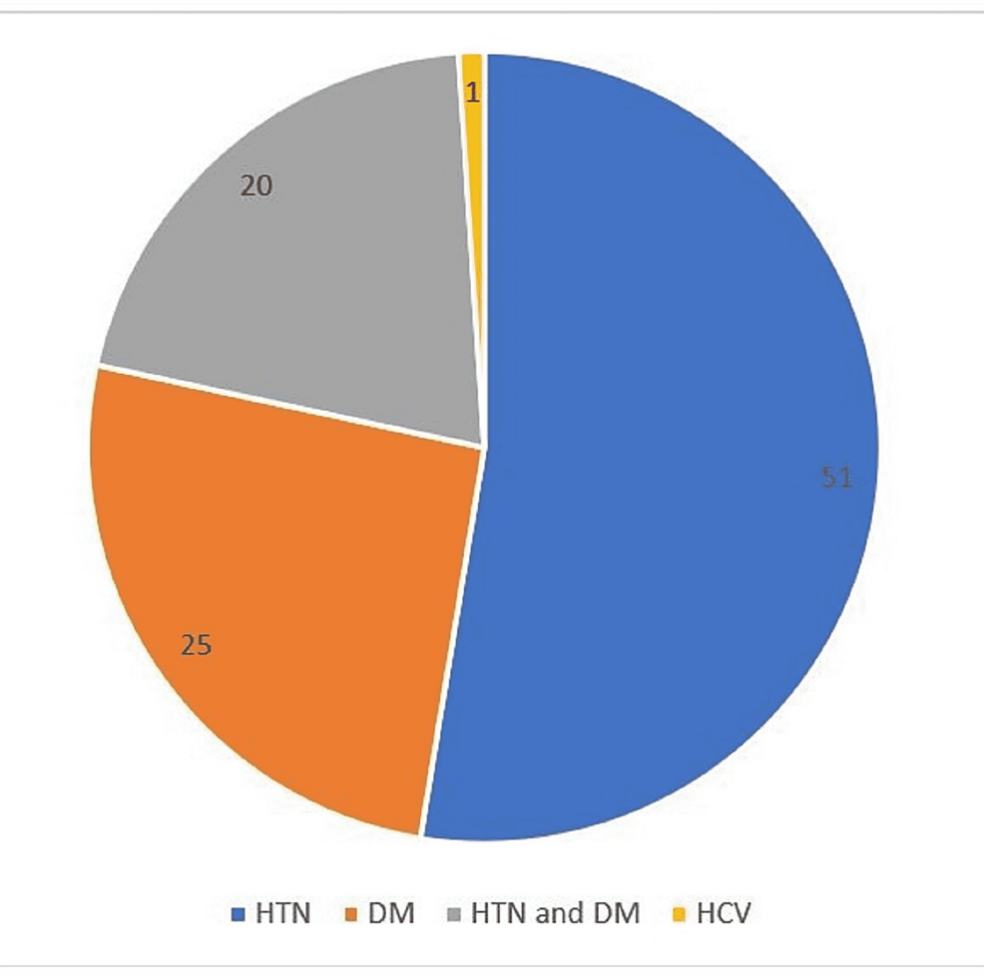
Associated co-morbidities in the post-KT patients. HTN = Hypertension, DM = Diabetes Mellitus, HCV = Hepatitis C Virus.

Eighty post-renal transplant patients with double J stents in-situ were recruited for this study with a mean age of 47.9 ± 12.1 years of which 60 (75%) were male. The associated co-morbidities are shown in figure 1.

There was no significant association between positive stent culture and patient age (p=0.562), sex (p=0.676) and diabetes (p=0.847). A majority (95%) of the patients had their stents removed at four weeks, and 2.5% had stents removed at two and eight weeks. There was a significant association between the duration of stent and stent colonization (p=0.031).

There was no organism cultured in both urine and stent in 13 (14.4%) patients. Nine (10%) patients had positive stent culture with negative urine culture, while 5 (5.6%) had positive urine culture with a negative stent culture. Fifty-eight (64.4%) of patients cultured the same organism from both urine and stent. Five patients (5.6%) had different organisms cultured in urine and stent (Tables 1, 2).

**Table 1:** The frequency of bacteria cultured in urine of post-KT patients.

Bacteria(urine)	Frequency(n=58)	Percent
E. coli	38	65.5
Klebsiella spp	10	17.2
Pseudomonas spp	6	10.4
Staphylococcus aureus	3	5.2
Coliform spp	1	1.7

**Table 2:** The frequency of bacteria cultured in stent of post-KT patients.

Bacteria(stent) growth	Frequency(n=62)	Percent
E. coli	36	58.1
Klebsiella spp	10	16.1
Pseudomonas spp	7	11.3
Staphylococcus aureus	4	6.5
Coliform spp	3	4.8
Streptococcus spp	2	3.2

**Figure 2:**
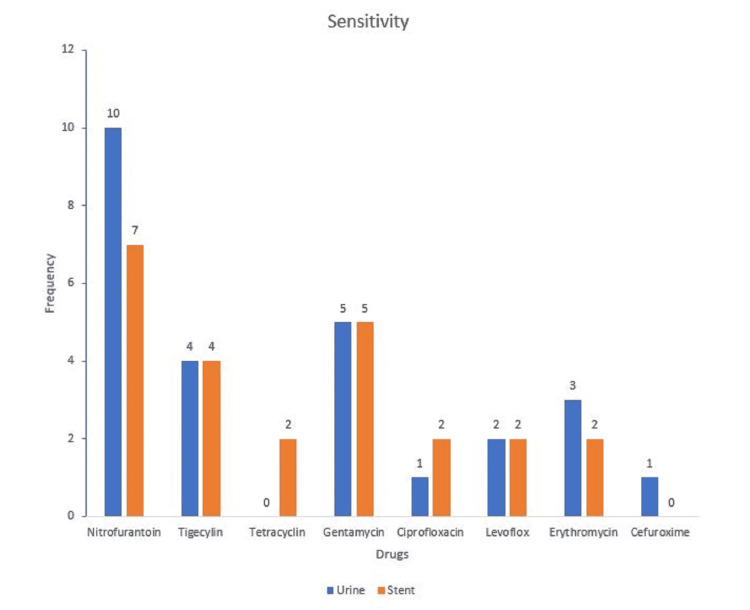
The sensitivity pattern of the bacteria cultured in urine and stent.

**Figure 3:**
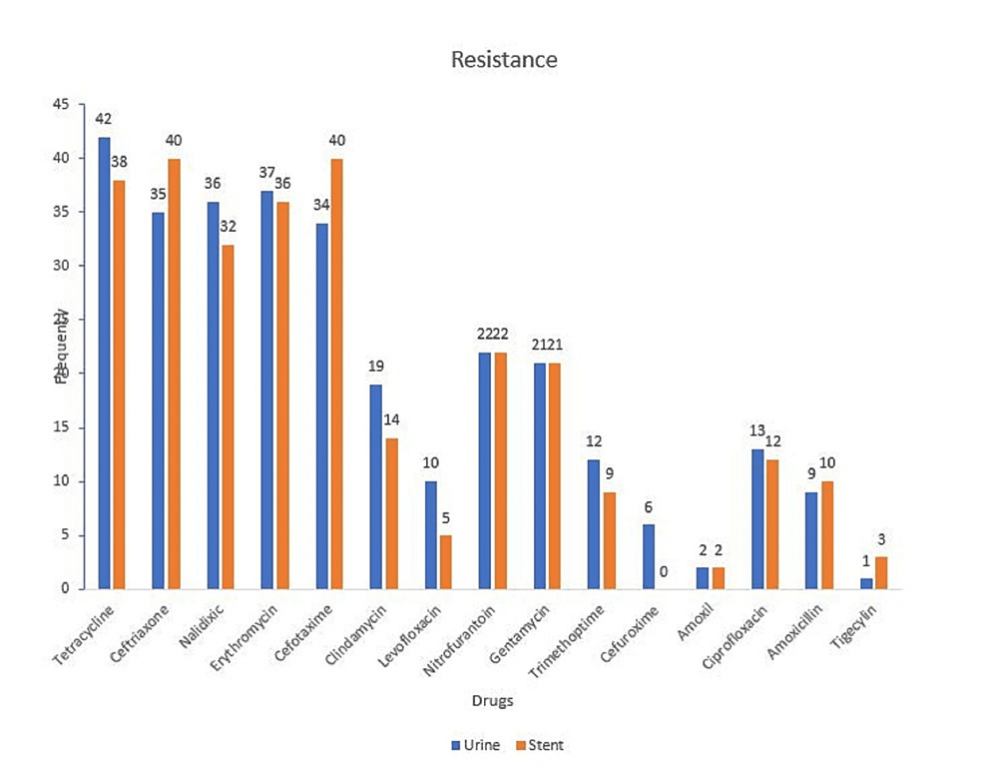
The resistance pattern of the bacteria cultured in urine and stent.

The sensitivity pattern shows that the organisms were more susceptible to nitrofurantoin and gentamicin, but resistant to tetracycline and ceftriaxone. Tigecycline showed good sensitivity and low resistance (Figure 2, 3).

## Discussion

Ureteric stenting for prophylaxis in renal transplant recipients is still a subject being debated [9]. It has the advantage of KTpreventing major surgical complications like ureteric leak and obstruction. However, it is not without its attendant complications such as UTI, hematuria, stent migration, stent encrustation, and forgotten stents [10, 11]. 

UTIs are the most common complications related to stents in patients following renal transplant [12]. Different studies have reported different frequencies of UTIs among kidney transplant recipients. Ranganathan et al. found that the incidence of UTI in patients with stents is significantly higher than in those without stents (71% vs 39%; p = 0.02) [13]. On the contrary, Mathe et al. found that the incidence of UTI in both groups was similar [stented (43.3%) vs non-stented (40.1%)] [14]. Derouich et al. reported that the occurrence of post-operative UTI is 47.2% in stented vs 48.7% in non-stented post-KT patients [15] and Shohab et al. showed there no significant difference in the occurrence of UTI between the two groups (40.5% vs 37.3%; p = 0.628) [7]. Post-operative UTI was found in 85.6% of the patients in the present study and this value is much higher than the values reported in the above studies. 

The mean age of 47.9 years in this study shows that ESRD is affecting the middle age population in sub-Sahara Africa with the majority of the affected population being male subjects. A similar study in Pakistan showed that the younger population is affected with a mean age 34.01 ± 14.63 years and the male population is more affected (75.2%) [7].

This study shows that hypertension and diabetes remain the most common co-morbidities associated with ESRD. In a single center study, Kehinde et al. revealed that the risk of bacteriuria and colonization of the J stent tip is increased significantly by a longer duration of stent retention, female gender, and systemic diseases such as diabetes mellitus, chronic renal failure, and diabetic nephropathy, and concluded that these categories of patients should undergo shorter stent retention, antimicrobial prophylaxis, and careful follow-up to minimize infectious complications [16]. The co-morbidity in patients in this study could contribute to the high frequency of urine and stent bacteria colonization. There was no association between age, sex, diabetes, duration of stents, and stent colonization in this study.

Two patients had their stents removed before four weeks post-kidney transplant because they did well postoperatively and there were concerns about access to endourological services for stent removal in their state of residence. Another two patients had their stents delayed for eight weeks because they had poor allograft function in which case, stents were left in place until allograft function improved.

Different strategies have been tried successfully to mitigate the infection complications by using different materials like silicone, polyethylene, polyurethane, biodegradable materials, and drug delivery materials, as well as coatings such as silver, heparin, polytetrafluoroethylene, phosphorylcholine biocides, or antibiotics [17]. A biofilm typically forms as a diverse population consisting of bacteria growing at different rates. The structure formed is encapsulated within a polymeric matrix that is self-developed and is able to adhere to different surfaces, biotic and abiotic. The use of systemic antibiotic therapy has proved futile in preventing colonization on the surfaces of materials, and this leaves the patient at continuous risk of infection and its possible attendant consequences [18-20].

A significant number of the subjects (64%) cultured the same organism in both urine and stent which shows similar bacteriology. The prevalence of stent without urine colonization in our study is almost double when compared to urine without stent colonization (10% versus 5.6%). All of these subjects were completely asymptomatic for UTIs. This is not unusual as bacteriuria without symptoms is frequently found in kidney transplant recipients, with an incidence close to 40% [21]. Recent evidence suggest that among kidney transplant recipients who are beyond the second month post-transplant, the prevalence of asymptomatic bacteriuria is low [22]. Some transplant centers in the Western World routinely screen for asymptomatic bacteriuria in post-transplant patients [23]. The usefulness of this strategy is debatable. There is inconclusive evidence to establish routine screening and antibiotic treatment in renal transplant recipients [24]. It is recommended by The American Society of Transplantation Infectious Diseases Guidelines to limit screening to the first month post-kidney transplant[25]. However, these are expert perspectives.

There is a large burden of evidence that asymptomatic bacteriuria does not affect long-term renal graft function prognosis [26-29]. A study by Klis et al. showed that urine and stents cultures are different in microscopy, sensitivity, and resistance. They concluded that while the risk of infection in the urine is low, the risk of bacterial colonization is high in patients that have the DJ catheter retained in their urinary tract [30]. 

E. coli was the most common organism cultured in both urine and stent accounting for 38% and 36% respectively [31]. Gram-negative microorganisms are the most common (around 50-90%) cause of UTI post kidney transplantation, with E. coli being the most commonly occurring microorganism found in urine cultures, and this is similar to findings in the general population [21]. The study in Nepal on post renal transplant patients also yielded E. coli as the most common organism isolated (64%) [32]. The high incidence of stent gram-negative organisms may indicate a preferential adhesion of these bacteria to the biomaterial surface. 

It has been discovered that Enterococcus spp. is the most important pathogen causing up to 30% of UTIs, especially in the first month following transplant [21]. In the following months, Enterococcus spp. is overtaken by E. coli as the commonest causative agent, this is closely followed in this order: Proteus spp., Klebsiella spp., Enterobacter spp., and Pseudomonas spp. [21]. This is similar to the findings in this study.

With the standard use of antimicrobial prophylaxis in post-renal transplant patients, uropathogenic bacteria are increasingly resistant to multiple antibiotics. The cultured organisms were most sensitive to nitrofurantoin which is not a commonly prescribed antibiotic and had poor sensitivity to cephalosporins and quinolones as shown in Figure 2. Cephalosporins showed very high resistance in this study as shown in Figure 3. In a study of post-renal transplant patients with asymptomatic bacteriuria caused by *E. coli*, samples from almost 80% of the patients were resistant to multiple antibiotics [33]. Tigecycline demonstrated good sensitivity and least resistance among the antibiotics tested in this study. This further illustrates that there is an increasing trend in resistance to commonly prescribed antibiotics.

Limitations

Our study was limited by its small sample size and its convenience sampling method. It is also limited by the prophylactic co-trimoxazole administration for Pneumocystic jerovenci pneumonia which may have affected the bacteria flora. Despite this, this study demonstrates the bacteriology of the colonization of DJ stents in clinical practice, providing data for surgeons to consider when deciding to use DJ stents. 

## Conclusions

This study shows that stent colonization was slightly higher than urine bacteriology with both demonstrating similar microbiological patterns. We found no significant association between stent colonization, sex, age, duration of the stent, and diabetes. There are no global patterns to the sensitivity and resistance of the uropathogens. Therefore, the initial empirical treatment of cases should be guided by finding from local studies. Initial therapy should be limited to a narrow spectrum of antibiotics pending the retrieval of culture and sensitivity results, after which an appropriate treatment regimen can be commenced depending on the outcome of the investigation. Moreover, antibiotic stewardship will help in reducing the trend of multidrug resistant pathogens.
